# Staple Formations in Bronchial Closure with Equal-Height Staples to Those with Graduated-Height Staples Using Motorized Staplers

**DOI:** 10.5761/atcs.oa.25-00031

**Published:** 2025-06-21

**Authors:** Kenji Tomizawa, Hana Oiki, Shota Fukuda, Masaya Nishino, Katsuaki Sato, Tetsuya Mitsudomi

**Affiliations:** 1 Division of Thoracic Surgery, Department of Surgery, Izumi City General Hospital, Izumi, Osaka, Japan; 2 Division of Thoracic Surgery, Department of Surgery, Faculty of Medicine, Kindai University, Osakasayama, Osaka, Japan

**Keywords:** staple formation, motorized stapler, bronchopulmonary fistula, lobectomy

## Abstract

**Purpose:** Motorized automatic staplers are used for bronchial closure following pulmonary resection. This study aimed to compare the completeness of staple formation in bronchial closure using 2 commonly adopted staple cartridges with motorized automatic staplers as follows: graduated-height staples (GHS) and equal-height staples (EHS).

**Methods:** This prospective observational study included 103 patients (105 bronchial stumps) undergoing pulmonary resections for lung cancer. Resected bronchi were embedded in paraffin, X-rays were taken, and staple formations were scored on a 0–4 scale, with a score of 4 indicating complete staple formation. Stump scores represented the average score of all staples per bronchial stump.

**Results:** The GHS exhibited a higher incidence of staple scores above the median (3.91) than that of the EHS (37/59 [62.7%] vs. 19/46 [41.3%], respectively; p = 0.033). Additionally, the GHS had a higher rate of complete staple formation than that in the EHS (84.7% vs. 75.1%; p <0.0001). This difference was more evident in calcified bronchi (84.2% vs. 57.6%, respectively; p <0.0001). No bronchopleural fistula was observed in any patients during the year.

**Conclusion:** Staple formations were generally more complete in the GHS than in the EHS. This difference was particularly notable in calcified bronchi.

## Introduction

**Fig. 1:**
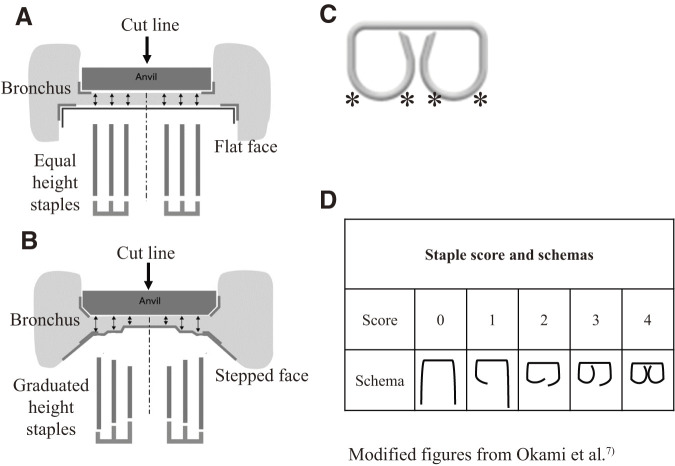
(**A**, **B**) Cartridge A features a flat face with 3 rows of EHS, while cartridge B has a stepped face with 3 rows of staples that progressively increase in height outward from the center cut line (GHS). (**C**, **D**) The scoring system employed a 5-point scale ranging from 0 to 4 to evaluate staple formations. The score assigned to each staple was determined based on the number of proper bends observed among the 4 bending points (^*^). A staple exhibiting 4 proper bending points received a score of 4. Panel (**D**) presents examples of staple scores and formations. EHS: equal-height staples; GHS: graduated-height staple

A bronchopleural fistula (BPF) is a potentially life-threatening postoperative complication of anatomic pulmonary resection,^1)^ the frequency of which reportedly varies between 1.3% and 4.4%.^2–5)^ In a Japanese study from 1962 to 1990, BPFs were observed in 52 of 2359 patients who underwent resection for lung cancer (2.1%). During this period, bronchial closure was predominantly achieved through manual suturing.^2)^ However, the introduction of manual automatic staplers has reduced the occurrence of BPFs compared to their occurrence in manual suturing. Another study conducted at the same institution, involving 553 patients undergoing pulmonary resection for lung cancer from 1995 to 1997, found BPFs in 7 patients (1.3%).^3)^ In this report, bronchial closure was performed using manual suturing in 50 (9%) patients and manual automatic staplers in 483 (91%), resulting in 4% and 1% BPF occurrence rates, respectively.^3)^ Moreover, improvements have been made to the cartridges with the increasing use of manual automatic staplers for bronchial closure. Currently, 2 types of cartridges are available for clinical use. One cartridge has the conventional design with a flat face and equal-height staples (EHS) (**Fig. 1A**), while the other features a stepped face with staples that rise progressively higher from the center-cut line (graduated-height staple [GHS]) (**Fig. 1B**). Medtronic (Minneapolis, MN, USA) introduced the GHS cartridge to the market in 2010. Assessment of these 2 cartridges using micro-computed tomography evaluation of blood flow at the gastric dissection margins in rats showed that the GHS cartridge exhibited superior blood flow than that in the EHS cartridge.^6)^ Therefore, preserving blood flow is crucial for closure and healing. Additionally, ensuring safer and more reliable bronchial closure is an important consideration for thoracic surgeons.

In 2017, a single-center prospective trial compared the staple formations of GHS and EHS cartridges in patients who underwent lobectomies using the Endo GIA Ultra Universal Stapler (Medtronic), a manual automatic stapler.^7)^ Staple formations were assessed on a scale of 0–4, with a score of 4 indicating complete staple formation. The percentage of complete staple formation was significantly higher in the GHS group than in the EHS group (25.3% vs. 10.0%). However, no BPFs were observed in any patients.

In current clinical practice, motorized automatic staplers have gained wider acceptance over manual ones. The first motorized automatic stapler, the iDrive powered stapling system, was introduced by Medtronic in 2010. Subsequently, motorized automatic staplers have undergone improvements and entered the market. In 2011, Ethicon (Cincinnati, OH, USA) introduced the Powered Echelon Flex, while Medtronic introduced the Signia stapling system in 2017. Additionally, Intuitive Surgical (Sunnyvale, CA, USA) launched the SureForm stapling system, specifically designed for robotic surgery using the da Vinci surgical system, in 2018. Notably, the development of these staplers has simplified surgical procedures and minimized technical disparities among surgeons. Motorized automatic staplers have significantly shorter operation times and lower blood loss, rates of postoperative pleurodesis, and hospital costs than those of manual staplers.^8)^

However, no comparison is currently available substantiating that motorized automatic staplers produce better staple formation than that produced by manual ones. Therefore, this prospective observational study aimed to evaluate the stapling ability of motorized automatic staplers for bronchial closure. We compared staple formations removed, intraoperative bleeding, and air leakage from the bronchial stumps, as well as BPF occurrence within 1year postoperatively, between the GHS and EHS groups.

## Materials and Methods

### Patients

We included patients aged ≥20 years who had undergone lobectomies, bi-lobectomies, or pneumonectomies at our institution. Patients with centrally located tumors, bronchoplasty, neoadjuvant therapy, pleural dissemination, malignant pleural effusion, or those who had undergone post-radiotherapy for the bronchus were excluded. Preoperative comorbidities were evaluated using the Charlson comorbidity index^9)^ and simplified comorbidity score.^10)^ Preoperative diabetic status was assessed using average blood sugar levels calculated from the preoperative glycated hemoglobin (HbA1c) values (equation: average blood sugar [ABS] = 28.7 × HbA1c − 46.7),^11)^ and preoperative nutritional status was assessed using the prognostic nutritional index.^12)^

### Surgical procedures and motorized automatic staplers

Pulmonary resections were performed using video-assisted thoracic surgery (VATS) or robotic-assisted thoracic surgery (RATS). VATS procedures utilized the Signia stapling system with GHS cartridges (Medtronic, while RATS procedures employed the SureForm stapling system with EHS cartridges (Intuitive Surgical). RATS was recommended for patients with preoperatively confirmed malignant tumors (EHS group), while VATS was performed if patients declined RATS or if no confirmed tissue diagnosis for malignancy was observed (GHS group). The Signia stapling system, a motorized automatic stapler used in the GHS group, measured tissue resistance values resulting from uneven tissue thickness and stiffness and automatically adjusted the stapling speed in 3 steps during the stapling and dissecting processes. Black cartridges were used in all patients in this group, with staple heights before and after formation ranging from 4.0 to 5.0 mm and 1.75 to 2.25 mm, respectively. In the EHS group, the SureForm stapling system used in the RATS measured tissue resistance, compressed the tissue to an appropriate thickness, and subsequently stapled and dissected it at a constant speed. If high tissue resistance was detected during stapling, the stapling system paused, compressed the tissue to an appropriate thickness, and subsequently resumed stapling and dissecting. The surgeon could select the black cartridge (staple height before and after formation: 4.6 and 2.3 mm, respectively) or the green cartridge (4.3 and 2.0 mm, respectively) at their discretion.

### Assessment of staple formations

Postoperatively, the bronchial stumps of the resected lungs were dissected along the staple lines into 3 sections. Next, the staple lines were embedded in paraffin and radiographed after formalin fixation. All staples were scored on a 5-point scale from 0 to 4, according to previous literature.^7)^ Well-functioning staples penetrated the tissue, with their 2 legs bending symmetrically at 4 points aligned with the anvil, as shown in **Fig. 1C**. The score is based on the number of proper bends at these 4 points. A staple with 4 proper bending points was assigned a score of 4. Examples of scores and staple formations from this study are presented in **Fig. 1D**. Staple scores were independently assessed by 4 blinded thoracic surgeons (K.T., K.S., M.N., and S.F.), and the average score was recorded as the staple score. Complete staple formation was defined as an average score of 4 points, and the average score of all staples in the bronchial stump was considered the stump score. The staple lines were defined as the inner, middle, and outer lines, in that order from the resected lung side, and the average staple score for each line was defined as the line score.

### Intraoperative and pathological evaluation of the bronchial stump

Intraoperative bleeding and air leakage (under an intra-airway pressure of 30 cmH_2_O) at the bronchial stump were evaluated. The calcification of the bronchial stump in the resected lung was examined microscopically. Additionally, the paraffin-embedded stapled bronchus thickness was measured using digital calipers.

### Statistical analysis

The chi-square or Fisher’s exact test was used to compare the percentage values among the groups. The t-test or Mann–Whitney U-test was used to compare the continuously distributed values among the groups. A 2-sided p-value <0.05 was considered statistically significant. All statistical analyses were performed using EZR (Saitama Medical Center, Jichi Medical University, Saitama, Japan), which is a graphical user interface for R (R Foundation for Statistical Computing, Vienna, Austria).^13)^

## Results

### Demographic and clinicopathological characteristics of the 103 patients

**Table 1** presents the clinicopathological characteristics of the study patients. Overall, 103 patients were included in the study (57 and 46 in the GHS and EHS groups, respectively). Among them, 97 patients had primary lung cancer. In the GHS group, 3 patients had pulmonary aspergillosis, whereas all patients in the EHS group had malignant tumors. However, no differences were observed in the various clinicopathological factors.

**Table 1:** Clinicopathological characteristics of 103 patients with pulmonary resection

Characteristics	GHS (n = 57)	EHS (n = 46)
Age, median (range)	73 (48–88)	73 (43–84)
Sex		
Male (%)	36 (63%)	24 (52%)
Smoking status		
Never (%)	37 (65%)	24 (52%)
BMI, average ± SD	22.8 ± 3.9	22.6 ±3.1
CCr, average ± SD	77.7 ± 27.4	71.6 ± 20.6
HbA1c, average ± SD	6.1 ± 0.9	6.1 ± 0.8
ABS, average ± SD	128.2 ± 26.6	127.4 ± 23.9
CCI, median (range)	1 (0–5)	1 (0–10)
SCS, median (range)	7 (0–17)	7 (0–18)
PNI, average ± SD	50.1 ± 4.7	51.1 ± 4.0
Primary disease		
Primary lung cancer	52 (91%)	45 (98%)
Metastatic lung tumor	2	1
Pulmonary aspergillosis	3	0
Mediastinal lymph node dissection		
Yes (%)	48 (84%)	42 (91%)
Operation time (minute), median (range)	178 (94–354)	176.5 (97–199)

GHS: graduated-height staples; EHS: equal-height staples; SD: standard deviation; BMI: body mass index; CCr: creatinine clearance; ABS: average blood sugar; CCI: Charlson comorbidity index; SCS: simplified comorbidity score; PNI: prognostic nutritional index; Hb: hemoglobin

### Evaluation and comparison of the bronchial stumps

In total, 105 bronchial stumps were evaluated, with 2 patients undergoing right upper and middle bi-lobectomies. **Table 2** presents the clinicopathological features of the 2 groups. Upper lobe bronchial stumps were the most common in both groups, followed by lower lobe bronchial stumps. In the GHS group, black cartridges were used for all 34 stumps, while black and green cartridges were used for 12 and 34 stumps, respectively, in the EHS group, at the surgeon's discretion. Pathological assessments revealed calcification in 15 and 9 bronchial stumps in the GHS and EHS groups, respectively. The stapled bronchus thickness was shorter in the GHS group than in the EHS group.

**Table 2:** Clinicopathological evaluation of 105 bronchial stumps

Characteristics	GHS (n = 59)	EHS (n = 46)
Tumor location		
Right (%)	41 (70%)	32 (70%)
Resected bronchus		
Upper bronchus	36	25
Middle bronchus	7	4
Lower bronchus	12	17
Intermediate trunk	3	0
Main bronchus	1	0
Cartridge color		
Black	59	34
Green	–^*^	12
Coverage with pericardial fat pad		
Yes (%)	14 (24%)	7 (15%)
Calcification		
Yes (%)	15 (25%)	9 (20%)
Thickness of stapled bronchus, average ± SD	2.06 ± 0.38	2.26 ± 0.46

^*^There is no standard for green cartridges in the GHS.

GHS: graduated-height staple; EHS: equal-height staple; SD: standard deviation

### Intraoperative bronchial assessments and postoperative BPF

Intraoperative air leakage from the bronchial stumps was observed in only 2 patients in the EHS group (4.3%), where black cartridges were used. Intraoperative bleeding from the bronchial stumps was significantly more frequent in the EHS group than in the GHS group (8/46 [17.4%] vs. 1/59 [1.7%], respectively; p = 0.009). However, no BPF was observed in any patients.

### Distribution of line score and stump score

**Fig. 2:**
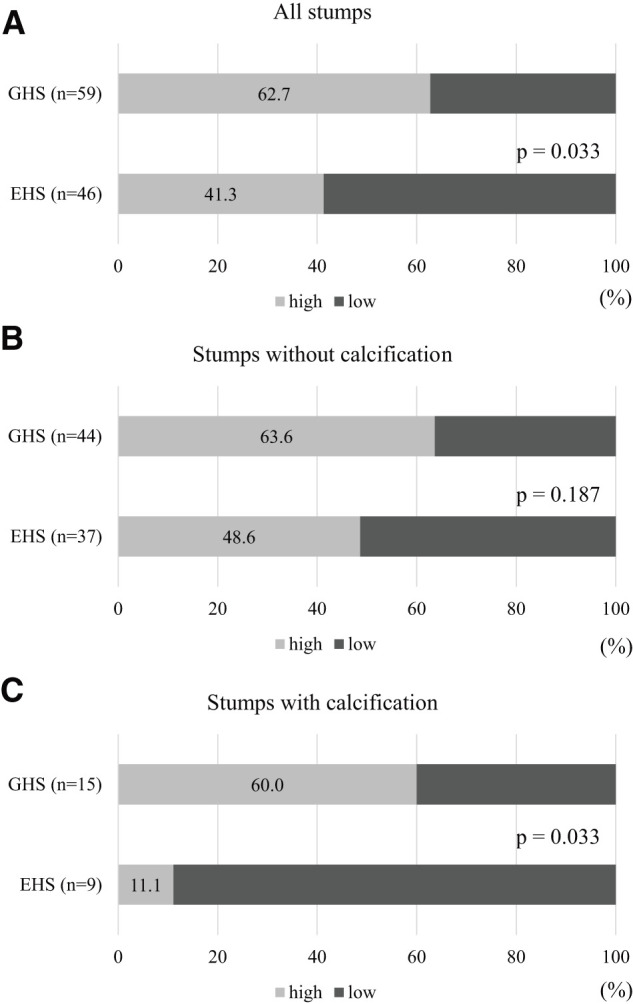
The median stump score was calculated as 3.91. In (**A**), stump scores of ≥3.91 were more predominantly observed in the GHS group than in the EHS group. Panels (**B**) and (**C**) present the distributions of stump scores, categorized according to the presence of calcification in the bronchial stumps. Although no significant differences were found in stump scores between the GHS and EHS groups for stumps without calcification, the GHS group exhibited significantly higher scores than those in the EHS group for stumps with calcification. GHS: graduated-height staple; EHS: equal-height staple

Only the inner line score was significantly higher in the GHS group (p = 0.047), with no significant differences between the middle and outer line scores (p = 0.190 and p = 0.139, respectively, data not shown). The median stump score for the 105 bronchial stumps was 3.91. Stump scores of ≥3.91 were more prevalent in the GHS group than in the EHS group (37/59 [62.7%] vs. 19/46 [41.3%], respectively; p = 0.033; **Fig. 2A**). The distribution of stump scores according to calcification of the bronchial stumps is presented in **Figs. 2B** and **2C**. No significant difference was observed in stump scores between the GHS and EHS groups without calcification (28/44 [63.6%] vs. 18/37 [48.6%], respectively; p = 0.187). However, stump scores for bronchial stumps with calcification were significantly higher in the GHS group than in the EHS group (9/15 [60.0%] vs. 1/9 [11.1%], respectively; p = 0.033).

### Distribution of staple scores

Overall, 1979 staples from 105 bronchial stumps were scored by 4 thoracic surgeons. Complete staple formations, with an average staple score of 4.00, were observed in 81.4% of the staples (1610/1979; **Table 3**). The rate of complete staple formation was significantly higher in the GHS group than in the EHS group (934/1103 [84.8%] vs. 658/876 [75.1%], respectively; p <0.0001). Regardless of calcification, the rate of complete staple formation was significantly higher in the GHS group than in the EHS group (without calcification: 711/838 [84.8%] vs. 559/704 [79.4%], respectively; p = 0.005; with calcification: 223/265 [84.2%] vs. 99/172 [57.6%], respectively; p <0.0001). In the GHS group, the rate of complete staple formation did not significantly differ between bronchial stumps with and without calcification (711/838 [84.8%] vs. 223/265 [84.2%], respectively; p = 0.770). However, it was significantly lower in bronchial stumps with calcification than in those without calcification in the EHS group (99/172 [57.6%] vs. 559/704 [79.4%], respectively; p <0.0001).

**Table 3:** Complete staple formation

	Total	GHS	EHS	p
Total stumps				
Staple number	1979	1103	876	<0.0001
Complete staple formation (%)	1610 (81.4%)	934 (84.7%)	658 (75.1%)	
Stumps without calcification				
Staple number	1542	838	704	0.0050
Complete staple formation (%)	1270 (82.4%)	711 (84.8%)	559 (79.4%)	
Stumps with calcification				
Staple number	437	265	172	<0.0001
Complete staple formation (%)	322 (73.7%)	223 (84.2%)	99 (57.6%)	

GHS: graduated-height staples; EHS: equal-height staples

## Discussion

We observed significantly higher stump scores in the GHS group than in the EHS group (p = 0.033). Moreover, the GHS group exhibited a significantly higher rate of complete staple formation than that in the EHS group (84.7% vs. 75.1%, respectively; p <0.0001). These findings are consistent with those of a previous study,^7)^ which reported complete staple formations in 24% and 10% of patients in the GHS and EHS groups, respectively. Although comparisons between studies are challenging due to variations in facilities and observers, the remarkable improvement in stapling ability achieved with motorized automatic staplers is noteworthy. Importantly, no patients with BPF were observed within 30 days postoperatively in either the GHS or EHS groups. Although long-term evaluation of bronchial closure with motorized automatic staplers was unavailable, these findings suggest that their use contributes to the prevention of short-term complications.

Several risk factors for BPF were reported: pneumonectomy, cancerous remnants at the bronchial stump, preoperative radiotherapy, and diabetes mellitus.^2)^ The frequency of BPF was 2.1% (52/2359), and this bronchial closure method was manual suturing.^2)^ With the clinical application of manual automatic staplers, the frequency of BPF decreased to 1%.^3)^ In this report, bronchial closure was performed using a motorized automatic stapler, and BPF was not observed. The method of bronchial closure may also be a risk factor for BPF. Recently, staple formation has been improved to be even better not only by automatic staplers but also by the development of cartridges. Staple formation allows a useful visual assessment of the status of bronchial closure. Although several risk factors have been involved in BPF, dysplasia of staple formation may be one of them. It is important to recognize as many risk factors as possible to further reduce the incidence of BPF, a less frequent but serious postoperative complication. Better staple formation may prevent BPF and provide reassurance to thoracic surgeons who manage bronchus, which is hard tissue.

The inner line score was significantly higher in the GHS group (p = 0.047). At the bronchotomy site, the bronchial tissues on the resected lung side are thickened due to the presence of segmental bronchial branch and lung parenchyma. The cartridge in the GHS group is progressively higher, suggesting that good staple formations are obtained for these bronchi. This may have led to the difference in staple scores between the GHS and EHS groups.

Two patients in the EHS group (4.3%) had intraoperative air leakage at the bronchial stumps in which black cartridges were used. The post-formed staple heights of the black and green cartridges were 2.3 and 2.0 mm, respectively. The average thickness of stapled bronchus was 2.26 ± 0.46 mm, and the post-formed staple of the black cartridge may have been too high in some patients. Conversely, no intraoperative air leakage of the bronchial stumps was noted in the GHS group, and the post-formed staple heights of the black cartridges were 1.75, 2.0, and 2.25 mm, respectively, from the outer to the inner line of the bronchial stump. There was a sufficient staple height to close the bronchus. In the EHS group, there were no patients with intraoperative air leakage after changing to a green cartridge, which had a post-formed staple height of 2.0 mm.

Calcification was detected in 14.2% of the bronchial stumps (15/106) in our study, whereas it was identified in 8.2% of bronchial stumps (5/61) in a study by Okami et al.,^7)^ although staple formation was not evaluated. Within the GHS group, no significant differences were found in the rate of complete staple formation between bronchial stumps with and without calcification. However, the EHS group exhibited a significantly lower rate of complete staple formation in bronchial stumps with calcification than that in those without calcification (p <0.0001). Notably, the GHS cartridge demonstrated excellent stapling ability in bronchial stumps with calcification.

This study has some limitations. First, adjusting for patient characteristics between the 2 groups was difficult because of insufficient prospective randomization. Second, using 2 motorized automatic staplers with different stapling systems and cartridges necessitates careful evaluation when comparing the GHS and EHS groups. For each cartridge, a comparison with a manual automatic stapler from the same manufacturer was preferred. However, there is no manual automatic stapler that matches the motorized one of the EHS group in the SureForm stapling system. Both groups have high rates of staple formation and are useful systems. Lastly, BPF incidence is extremely low, making it difficult to establish a causal relationship between staple dysplasia and BPF occurrence.

## Conclusions

Our study shows that staple formations were generally more complete in the GHS group than in the EHS group. This difference was particularly notable in calcified bronchi. Although no difference was observed in early postoperative bronchial fistula incidence, caution and extended follow-up are advised, since calcification of the bronchi is frequently difficult to detect before pathologic evaluation.

## Acknowledgments

We thank Dr. Sano from the Pathology Department, Izumi City General Hospital, for evaluating the bronchial stumps for calcification, and Mr. Nakamura from the Pathology Department, Izumi City General Hospital, for cutting the staple lines and embedding them in paraffin. We would like to thank Editage (www.editage.com) for English language editing.

## Declarations

### Ethics approval and consent to participate

This study was reviewed and approved by the Ethics Committee of Izumi City General Hospital (21-J05-01). The study was conducted in accordance with the ethical standards of the Declaration of Helsinki of 1975.

### Consent for publication

Written informed consent was obtained from all eligible patients between June 2021 and September 2022.

### Funding

The authors have no funding sources to disclose.

### Data availability

Not applicable.

### Author contributions

Kenji Tomizawa designed this study and analyzed the data. Kenji Tomizawa and Tetsuya Mitsudomi prepared the figures and wrote the original draft. Hana Oiki, Shota Fukuda, Masaya Nishino, and Katsuaki Sato oversaw the study and revised the article. All authors reviewed and approved the final manuscript.

### Disclosure statement

The authors have no conflicts of interest to disclose.
